# Supplementary material for: Health and well-being of trans and non-binary participants in a community-based survey of gay, bisexual, and queer men, and non-binary and Two-Spirit people across Canada
Source: PLoS One. 2021 Feb 11;16(2):e0246525. doi: 10.1371/journal.pone.0246525 (PMC7877578; doi:10.1371/journal.pone.0246525)
Supplement: Attachment — Submitted filename: ResponsetoReviewers.docx [file pone.0246525.s001.docx]

**RESPONSE TO REVIEWERS**

**Thank you for the opportunity to revise and resubmit this manuscript for consideration in *PLOS ONE*. We are appreciative of the comments provided by the editor and reviewers, which have strengthened this manuscript. Authors responses are in bullet-points and bolded text below reviewer comments.**

**We would like to amend our Financial Disclosure statement to the following:**

**Sex Now 2018 received funding support from Canadian Blood Services MSM Research Fund, funded by the federal government (Health Canada) and the provincial and territorial ministries of health. The views herein do not necessarily reflect the views of Canadian Blood Services or the federal, provincial, or territorial governments of Canada. Additional in-kind contributions were received from the Public Health Agency of Canada's National Microbiology Laboratory. Additional funding for this analysis was provided by Women and Gender Equality (WAGE) Canada. NJL is supported by a Scholar Award from the Michael Smith Foundation for Health Research (#16863).**

When submitting your revision, we need you to address these additional requirements. Please ensure that your manuscript meets PLOS ONE's style requirements, including those for file naming. The PLOS ONE style templates can be found at https://journals.plos.org/plosone/s/file?id=wjVg/PLOSOne_formatting_sample_main_body.pdf and <https://journals.plos.org/plosone/s/file?id=ba62/PLOSOne_formatting_sample_title_authors_affiliations.pdf>

- **We have made changes to the manuscript based on the provided submission guidelines.**

Please state in your methods section whether you obtained consent from parents or guardians of the minors included in the study or whether the research ethics committee or IRB approved the lack of parent or guardian consent.

- **Thank you. We have clarified that no parental consent was required per our REB approval in lines 180 and 181 of the clean manuscript.**

We note that you have indicated that data from this study are available upon request. PLOS only allows data to be available upon request if there are legal or ethical restrictions on sharing data publicly. For information on unacceptable data access restrictions, please see http://journals.plos.org/plosone/s/data-availability#loc-unacceptable-data-access-restrictions. In your revised cover letter, please address the following prompts: a) If there are ethical or legal restrictions on sharing a de-identified data set, please explain them in detail (e.g., data contain potentially identifying or sensitive patient information) and who has imposed them (e.g., an ethics committee). Please also provide contact information for a data access committee, ethics committee, or other institutional body to which data requests may be sent.

If there are no restrictions, please upload the minimal anonymized data set necessary to replicate your study findings as either Supporting Information files or to a stable, public repository and provide us with the relevant URLs, DOIs, or accession numbers.

Please see http://www.bmj.com/content/340/bmj.c181.long for guidelines on how to de-identify and prepare clinical data for publication. For a list of acceptable repositories, please see <http://journals.plos.org/plosone/s/data-availability#locrecommended-repositories>."

- **The University of Victoria's Human Research Ethics Board has only approved storage of our data on secure university servers since the data contain potentially sensitive information about study participants. Data is available on request through secure university servers only. Any requests to access the data can be made to University of Victoria's Human Research Ethics Board (250-472-4545 or** [**ethics@uvic.ca**](mailto:ethics@uvic.ca)**).**

*Reviewers' comments: Reviewer's Responses to Questions Comments to the Author 1.*

Is the manuscript technically sound, and do the data support the conclusions? The manuscript must describe a technically sound piece of scientific research with data that supports the conclusions. Experiments must have been conducted rigorously, with appropriate controls, replication, and sample sizes. The conclusions must be drawn appropriately based on the data presented. Reviewer #1: Yes Reviewer #2: Yes

- **Response Not Applicable**

Has the statistical analysis been performed appropriately and rigorously? Reviewer #1: Yes Reviewer #2: Yes

- **Response Not Applicable**

Have the authors made all data underlying the findings in their manuscript fully available? The PLOS Data policy requires authors to make all data underlying the findings described in their manuscript fully available without restriction, with rare exception (please refer to the Data Availability Statement in the manuscript PDF file). The data should be provided as part of the manuscript or its supporting information, or deposited to a public repository. For example, in addition to summary statistics, the data points behind means, medians and variance measures should be available. If there are restrictions on publicly sharing data—e.g. participant privacy or use of data from a third party—those must be specified. Reviewer #1: No Reviewer #2: No

- **The University of Victoria's Human Research Ethics Board has only approved storage of our data on secure university servers since the data contain potentially sensitive information about study participants. Data is available on request through secure university servers only. Any requests to access the data can be made to Dr. Nathan Lachowsky at the University of Victoria (nlachowsky@uvic.ca).**

Is the manuscript presented in an intelligible fashion and written in standard English? PLOS ONE does not copyedit accepted manuscripts, so the language in submitted articles must be clear, correct, and unambiguous. Any typographical or grammatical errors should be corrected at revision, so please note any specific errors here. Reviewer #1: Yes Reviewer #2: Yes

- **Response Not Applicable**

*Review Comments to the Author Please use the space provided to explain your answers to the questions above. You may also include additional comments for the author, including concerns about dual publication, research ethics, or publication ethics. (Please upload your review as an attachment if it exceeds 20,000 characters)*

If possible (word count permitting), could you make it more clear in the abstract that cisgender women were the only potential participants excluded based on gender?

- **Line 27 includes a sentence clarifying that all people who identified as women were ineligible**

In the methods section, could you provide the thresholds/criteria used to determine statistical significance?

- **Thank you. We have added text in the methods section to provide this information in lines 214-215 of the clean manuscript.**

In the results section, I'm assuming you mean "statistically significant" when you say significant differences?

- **Correct. We have added qualifiers in throughout results to clarify we mean “statistically significant.”**

For the adjusted ORs (starting with Table 2) in each health topic area (e.g. mental health, sub use, STIs, etc.), were each of these variables assessed in separate models? Each category has an AOR, but from the methods section it sounds like there were only 5 confounders (so I'm assuming they weren't all in the same model or even topic-based models). So were these 5 confounders used for ALL of these variables of interest? More clarification in the model construction would help. That's all my comments. Really well-written and well done.

- **We have added content in lines 212-213 and 290-291 to clarify that each variable was assessed in separate models, and adjusted for the same 5 confounders.**

*General - Terminology:*

Authors use various terminology to describe sexual minority men. In row 40, I would introduce ‘sexual minority men’ as an umbrella term.

- **Thank you. We have added the phrase ‘sexual minority’ men and replaced instances of ‘minoritized’ with ‘minority’.**

Authors use language ‘minoritized’ to describes sexual and gender minority people. Highly recommend the authors modify the language from ‘minoritized’ to ‘minority’, which is consistent with the current literature.

- **See comment directly above.**

I would strongly recommend some additional context for the designation of the ‘non-binary’ group. Authors categorized participants in this group who responded “neither” to the question “What is your gender identity?” with the possible responses being: “man” “woman” or “neither. I prefer to self-describe as: _____________.” From my reading of the article, authors should define the term ‘non-binary’ and its rationale for use based on the response options provided above. Since ‘non-binary’ is an identity label, it is very possible that participants self-described their identity as non-binary in the open text field. But for those participants who didn’t, it needs to be clear that its use in this article is more of an umbrella term.

- **We have added content in rows 197-200 to give context and rationale for the non-binary group.**

Abstract: Row 32: Please add comparison group (cisgender sexual minority men)

- **We have added the comparison group**.

Introduction: Row 49: Authors provided an example of a healthcare disparity – I would suggest changing the term ‘health disparities’ to ‘healthcare disparities’.

- **We have changed the term to healthcare disparities.**

Row 64: Health disparities are introduced here. I would recommend modifying this sentence: “Additionally, trans and non- binary people experience health disparities, and report poorer physical and mental health than their cisgender peers”.

- **We have edited this sentence as suggested.**

Row 84: Recommend changing “statistics” to “findings”. Row 84: It is unclear if these findings are associated with the Trans PULSE study or the qualitative interviews conducted by Rich et al.: “Some participants were living in an urban centre and well-connected to healthcare systems, such as routine transition-related care appointments that made integrating HIV testing into their regular healthcare regimen easy”. Please clarify.

- **We have changed ‘statistics’ to ‘findings’ and clarified that findings referenced the Rich et al study.**

Row 98: I would recommend clarifying the time-period for ‘did not have enough to eat’

- **We have clarified that this was asked for a 12-month period.**

Row 155: I would recommend moving the social media recruitment efforts earlier in the paragraph with the other recruitment efforts.

- **We have moved this earlier in the paragraph.**

Results: Due to the questions about gender identity and trans experience, the authors indicated they are unable to provide statistical comparisons between the those who identified as trans and those who were categorized in the non-binary group and acknowledge this as a limitation. I would suggest that the authors discuss rationale for not using an analysis plan with four categories: (1) cisgender, (2) trans, (3) non-binary, and (4) non-binary with trans experience.

- **We have added clarification and rationale for our gender groupings in the results section (lines 577-582 of the clean manuscript).**
